# Supplementary material for: AAV-mediated peripheral single chain variable fragments’ administration to reduce cerebral tau in adult P301S transgenic mice: mono- vs combination therapy
Source: bioRxiv. 2025 Feb 17:2025.02.13.638144. Preprint. [Version 1] doi: 10.1101/2025.02.13.638144 (PMC11870445; doi:10.1101/2025.02.13.638144)
Supplement: Supplement 1 [file NIHPP2025.02.13.638144v1-supplement-1.pdf]

bioRxiv preprint doi: https://doi.org/10.1101/2025.02.13.638144; this version posted February 17, 2025. The copyright holder for this preprint (which was not certified by peer review) is the author/funder, who has granted bioRxiv a license to display the preprint in perpetuity. It is made available under aCC-BY 4.0 International license.

871 **Supplementary figure 1**

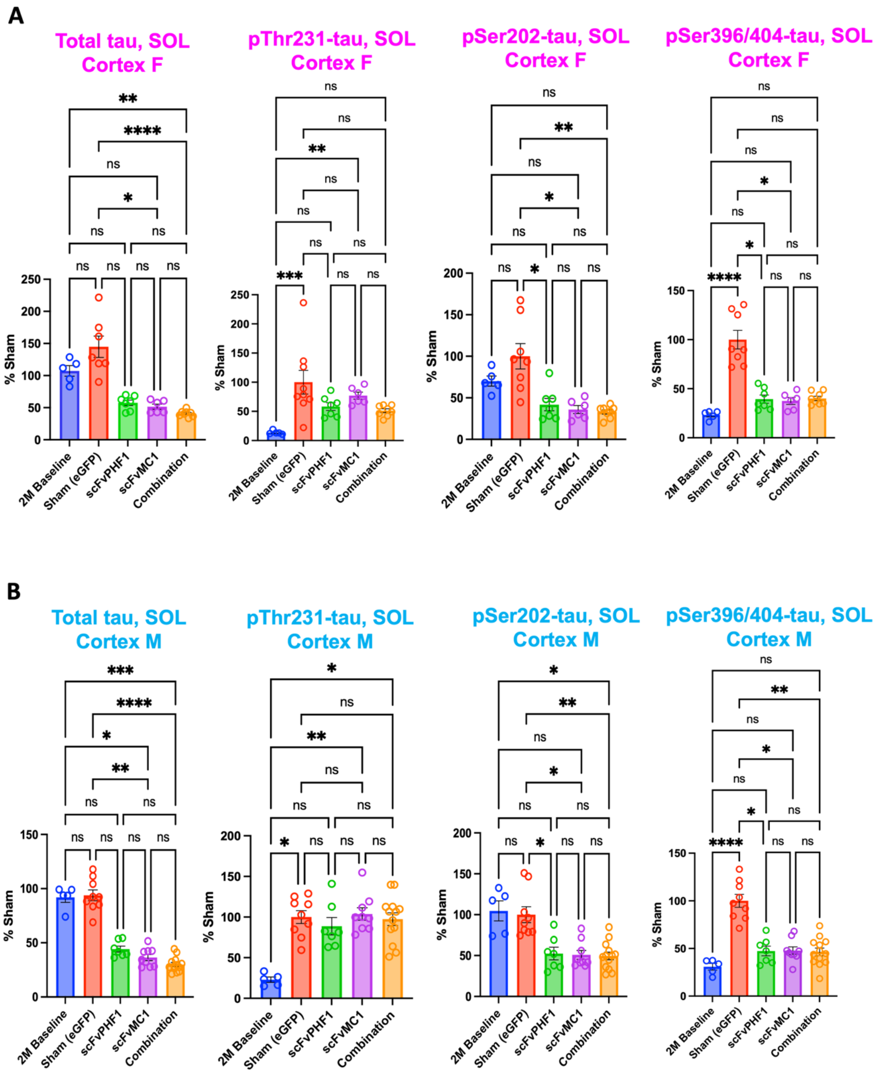

Figure S1. scFv-MC1 reduces soluble tau in the cortex without major sex differences, related to Figure 2. (A) ELISA quantifications of soluble (SOL) total tau, pThr231-tau, pSer202-tau, and pSer396/-404-tau in females: baseline (n = 5), sham-eGFP (n = 9), scFv-PHF1 (n = 7), scFv-MC1 (n = 6), and combination (n = 8). **(B)** ELISA quantifications of soluble (SOL) total tau, pThr231-tau, pSer202-tau, and pSer396/-404-tau in males: baseline (n = 5), sham-eGFP (n = 9), scFv-PHF1 (n = 7), scFv-MC1 (n = 9), and combination (n = 13). Data were normalized to the percent sham-eGFP group and represent mean ± SEM. Statistical analysis was performed by parametric Kruskal-Wallis test. * $P < 0.05$; ** $P < 0.01$; *** $P < 0.001$; **** $P < 0.0001$.

872

bioRxiv preprint doi: https://doi.org/10.1101/2025.02.13.638144; this version posted February 17, 2025. The copyright holder for this preprint (which was not certified by peer review) is the author/funder, who has granted bioRxiv a license to display the preprint in perpetuity. It is made available under aCC-BY 4.0 International license.

Supplementary figure 2

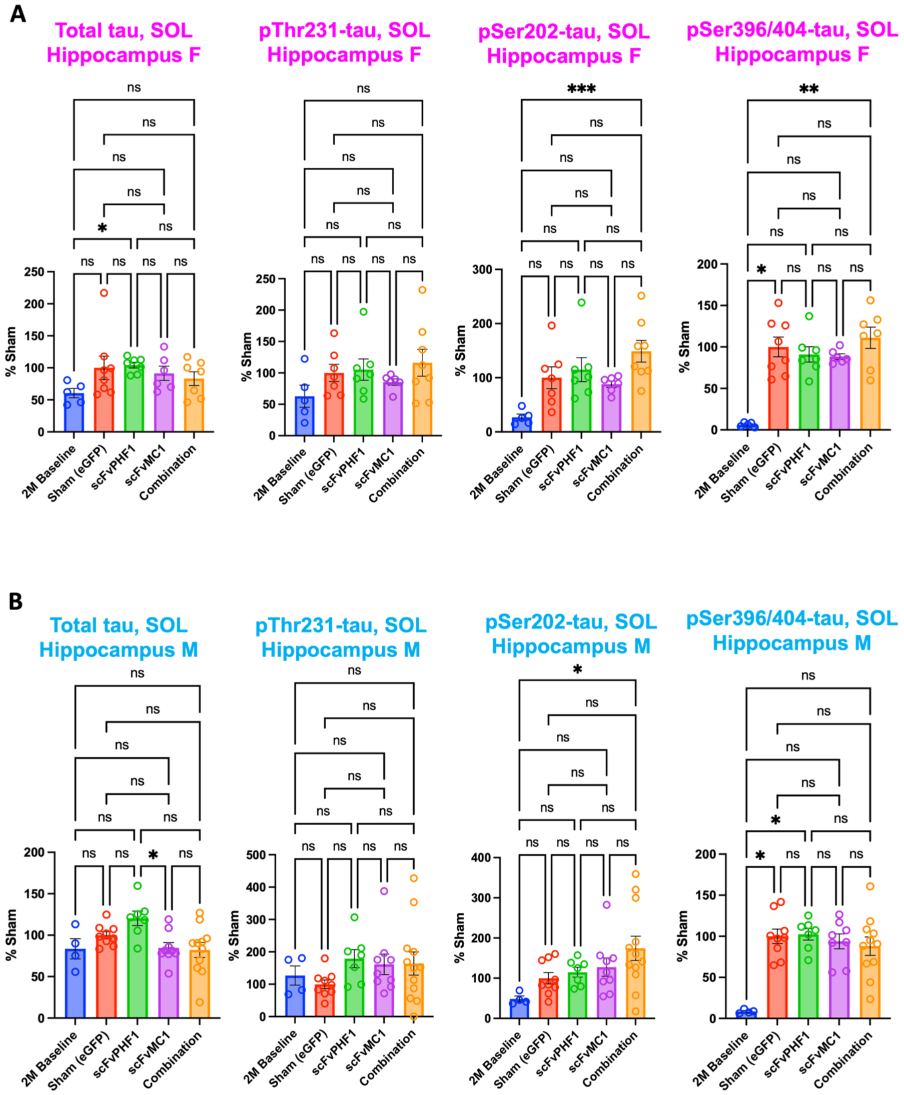

Figure S2. Hippocampal soluble tau remains relatively unchanged across sexes, related to Figure 3. (A) ELISA quantifications of soluble (SOL) total tau, pThr231-tau, pSer202-tau, and pSer396/-404-tau in females: baseline (n = 5), sham-eGFP (n = 8), scFv-PHF1 (n = 7), scFv-MC1 (n = 6), and combination (n = 8). **(B)** ELISA quantifications of soluble (SOL) male total tau, pThr231-tau, pSer202-tau, and pSer396/-404-tau in males: baseline (n = 5), sham-eGFP (n = 9), scFv-PHF1 (n = 9), scFv-MC1 (n = 7), and combination (n = 12). Data were normalized to the percent sham-eGFP group and represent mean ± SEM. Statistical analysis was performed by parametric Kruskal-Wallis test. * $P < 0.05$; ** $P < 0.01$; *** $P < 0.001$.

bioRxiv preprint doi: https://doi.org/10.1101/2025.02.13.638144; this version posted February 17, 2025. The copyright holder for this preprint (which was not certified by peer review) is the author/funder, who has granted bioRxiv a license to display the preprint in perpetuity. It is made available under aCC-BY 4.0 International license.

Supplementary figure 3

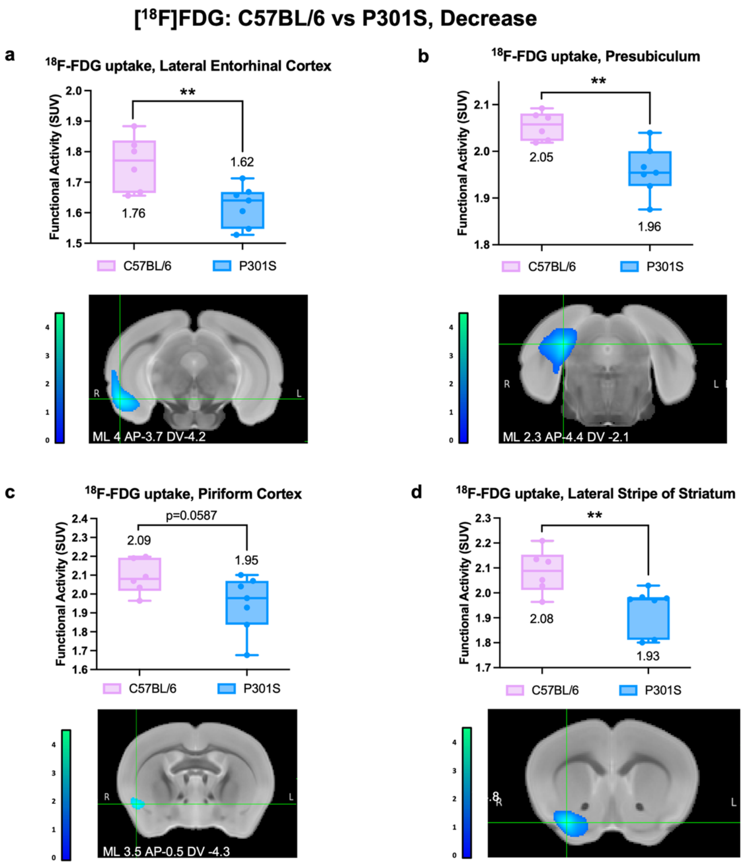

e

Decrease in ^{18}FDG uptake
P **<0.05, Cluster cutoff k=400**

Brain Region	ML	AP	DV	Functional Activity (SUV)	Cluster Voxels
Lateral Entorhinal Cortex	4	-3.7	-4.2	C57/BL6 = 1.76<br>P301S =1.62	8113
Presubiculum	2.3	-4.4	-2.1	C57/BL6 = 2.05<br>P301S = 1.96	10592
Piriform Cortex	3.5	-0.5	-4.3	C57/BL6 = 2.09<br>P301S =1.95	530
Lateral Stripe of Striatum	2.1	1	-4.8	C57/BL6 = 2.08<br>P301S = 1.93	5544

Fig S3. Brain regions with decreased ^{18}F-FDG uptake in C57BL/6 vs P301S mice, related to Figure 7. Coronal view of statistically significant decrease C57 BL/6 mice (n=6) vs. P301S (n=7) in relative ^{18}F-FDG uptake at P < 0.05. Regions with lower metabolic activity includes lateral entorhinal cortex **(a)**,

bioRxiv preprint doi: https://doi.org/10.1101/2025.02.13.638144; this version posted February 17, 2025. The copyright holder for this preprint (which was not certified by peer review) is the author/funder, who has granted bioRxiv a license to display the preprint in perpetuity. It is made available under aCC-BY 4.0 International license.

presubiculum **(b)**, piriform cortex **(c)**, and lateral stripe of striatum **(d)**. Statistically significant clusters were extracted using FSLeyes and overlaid onto coronal view mouse brain MRI template, Paxinos & Franklin anatomical coordinates shown. Color bar representation of ^{18}F-FDG decrease threshold value. Data are expressed as the mean + SEM. * $P < 0.05$, ** $P < 0.01$, *** $P < 0.001$, two tailed unpaired t-test with Welch's correction. **(e)** Brain regions in Paxinos and Franklin coordinates for significant clusters of ^{18}FDG activation: table includes stereotactic coordinates: ML (mediolateral), AP (anterior posterior), DV (dorsoventral), functional activity average in C57/BL6 and P301S, and cluster voxels. SPM derived cluster value (standardized uptake values, SUV), P <0.05, Cluster cutoff k=400.

bioRxiv preprint doi: https://doi.org/10.1101/2025.02.13.638144; this version posted February 17, 2025. The copyright holder for this preprint (which was not certified by peer review) is the author/funder, who has granted bioRxiv a license to display the preprint in perpetuity. It is made available under aCC-BY 4.0 International license.

879 **Supplementary figure 4**

A

Increase in ^{18}FDG uptake
***P* <0.05, Cluster cutoff k=50**

Brain Region	ML	AP	DV	Functional Activity (SUV)	Cluster size (Voxels)
Dentate Gyrus	1.6	-3.1	-2.2	Sham = 2.00<br>scFv MC1 =2.07	1323
Entopeduncular Nucleus	-1.8	-1.3	-4.3	Sham = 1.94<br>scFv MC1 =2.01	1275
Reticular Nucleus	-1	-8.5	-5.4	Sham = 1.30<br>scFv MC1 =1.40	549
Periaqueductal Gray	0.4	-4.2	-3.1	Sham = 2.18<br>scFv MC1 =2.33	2450

B

Decrease in ^{18}FDG uptake
***P* <0.01, Cluster cutoff k=100**

Brain Region	ML	AP	DV	Functional Activity (SUV)	Cluster Voxels
Amygdala	-1.9	-1.1	-5.9	Sham = 0.63<br>scFv MC1 =0.53	11244

Figure S4. ^{18}F-FDG uptake: brain regions, functional activity average and cluster voxels in P301S treated and sham-eGFP mice, related to Figure 7. (A) Brain regions in Paxinos and Franklin coordinates for significant clusters of ^{18}FDG activation. Increase in ^{18}FDG uptake in scFvMC1 treated mice. SPM group comparison in Sham (n=7) vs scFv MC1 (n=7), SPM derived cluster value (standardized uptake values, SUV), p<0.05 with voxel cutoff at k=50. **(B)** Brain regions in Paxinos and Franklin coordinates for significant clusters of ^{18}FDG activation. Decrease in ^{18}FDG uptake in scFvMC1 mice. SPM group comparison in Sham, (n=7) vs scFv MC1 (n=7), SPM derived cluster value (standardized uptake values, SUV), p<0.01, Cluster cutoff k=100.
ML (mediolateral), AP (anterior posterior), DV (dorsoventral).

880
